# Growth and Development Status of Children Post–COVID-19 Infection: Cross-Sectional Questionnaire Study

**DOI:** 10.2196/75057

**Published:** 2025-07-29

**Authors:** Herlina Febrianti, Dessie Wanda, Efa Apriyanti

**Affiliations:** 1 Faculty of Nursing, Universitas Indonesia, Kampus UI Depok Jawa Barat, Depok, 16424, Indonesia, 62 2178849120, 62 217864124

**Keywords:** child, COVID-19, growth, development, developmental status

## Abstract

**Background:**

COVID-19 may impact children’s growth and development, potentially leading to various health issues.

**Objective:**

This study aimed to identify factors associated with the growth and development status of children under 5 years of age after COVID-19 infection.

**Methods:**

This cross-sectional study included 292 children under five years of age assessed after COVID-19 infection. All participants had negative results via polymerase chain reaction (PCR) test and were hospitalized at a type A hospital in Jakarta between July 2021 and December 2022. Participants were selected using purposive sampling techniques.

**Results:**

Male sex and the age group of 25‐36 months were significantly associated with growth status. The most dominant factor associated with child development was the presence of comorbidities.

**Conclusions:**

This study recommends improving hospital discharge planning and strengthening community health services to support children’s growth and development after discharge.

## Introduction

COVID-19 in children is generally milder than in adults [1], although comorbidities can worsen outcomes. Data indicate that 50.9% of children had mild symptoms, and 38.3% experienced moderate symptoms, with infants being the most likely to develop acute or critical illness, followed by preschool-aged children [2]. Beyond the direct health effects, the pandemic has also posed significant threats to children’s growth and development.

COVID-19 has affected various developmental domains, including language [3,4], independence, and motor skills [4]. Growth and developmental trajectories are influenced by gender across all ages [5]; during the pandemic, gender also shaped behavioral responses, with adolescent girls particularly prone to depression and loneliness [6-8].

Lockdown measures contributed to increased screen time among children and adolescents [9,10], limiting social interactions and increasing risks of acute stress disorder, anxiety, and depression [11-14]. Isolation also led to weight gain due to reduced physical activity and overeating [4,9,11].

To date, no studies have comprehensively addressed the effects of COVID-19 on children’s growth and development. This study aims to identify factors associated with post–COVID-19 growth and development in children.

## Methods

### Study Design

This was an analytical cross-sectional study.

### Sample

The study sample included 292 children selected with the purposive sampling method. Inclusion criteria were: under five years of age, three months post–COVID-19 infection, negative PCR test results, and hospitalization for COVID-19 between July 2021 and December 2022. If a respondent had passed away or their parents declined participation, they were excluded from the study.

### Instrument

The instruments used included a demographic questionnaire and the Prescreening Developmental Questionnaire (PDQ), based on age groups.

### Data Collection

Respondent data were retrieved from the medical records of a top referral hospital in Jakarta. Addresses were provided to enumerators for direct data collection at each respondent’s home. This study involved 15 enumerators, who underwent training sessions covering PDQ administration, weight and height measurements. Inter-rater reliability was assessed using Cohen κ, with values ranging from 0.61 to 1. The lowest κ value recorded among the enumerators was 0.783, indicating a high degree of consistency between the researcher and enumerators.

### Data Analysis

The data were analyzed using SPSS software (version 25.0; IBM Corp). Normality testing was conducted using the Kolmogorov-Smirnov test for numerical data, which yielded *P*<.05, indicating non-normal distribution. Bivariate analysis of categorical data was performed using the *χ*^2^ test. Numerical-categorical data were analyzed using the Mann-Whitney test. Multivariate analysis was conducted by multiple logistic regression.

### Ethical Considerations

Ethical approval was obtained from the Fakultas Kedokteran Universitas Indonesia (FKUI) - Cipto Mangunkusumo Hospital Ethical Committee (approval number: KET-668/UN2.F1/ETIK/PPM.00.02/2023). Informed consent was obtained from the parents, and all patients’ identities were kept confidential. To protect participant privacy and confidentiality, all personal identifiers were removed during data collection, and the data were fully anonymized before analysis. Access to the raw data was restricted to authorized research personnel only. Participation in the study was entirely voluntary, and no compensation was offered for involvement.

## Results

The characteristics of the respondents and their growth and developmental status post–COVID-19 infection are presented in Table 1.

**Table 1.** The distribution of children’s characteristics and growth and developmental status post–COVID-19 infection.

Variables	Respondents (N=292)
Age (months), n (%)	
49‐60	51 (17.5)
37‐48	64 (21.9)
25‐36	88 (30.1)
12‐24	89 (30.5)
Gender, n (%)	
Girl	132 (45.2)
Boy	160 (54.8)
Comorbidity, n (%)	
No	120 (41.1)
Yes	172 (58.9)
Severity, n (%)	
Asymptomatic	16 (5.5)
Mild – moderate	235 (80.5)
Severe – critical	41 (14)
Length of stay (day), median (IQR)	3 (1-28)
Growth (weight-for-age), n (%)	
Normal weight	186 (63.7)
Underweight	106 (36.3)
Growth (length/height-for-age), n (%)	
Normal stature	157 (53.8)
Short stature	135 (46.2)
Development, n (%)	
Normal or typical	100 (34.2)
Atypical	192 (65.8)

Most respondents had normal weight-for-age (n=186, 63.7%) and length/height-for-age (n=157, 53.8%). However, 192 (65.8%) of respondents identified as having a potential health concern classified as atypical according to the PDQ.

Further analysis was conducted to identify the correlation between physiological factors (ie, age, gender, severity, comorbidities) as well as situational factors (eg, length of stay) and children’s growth and developmental status. As shown in Table 2, underweight status was the most common among children aged 25‐36 months (n=43, 48.9%), male sex (n=60, 37.5%), no comorbidities (n=46, 38.3%), and with severe to critical illness (n=18, 43.9%); only age was significantly associated with weight-for-age growth (*P*<.05).

**Table 2.** The correlation between physiological and situational factors with children’s weight-for-age post–COVID-19 (n=292).

Variables	Respondents (N=292), n	Growth status			
		Normal weight (n=186)	Underweight (n=106)	OR (95% CI)	*P* value
Age (months), n (%)					
49‐60	51	43 (84.3)	8 (15.7)	Ref	—^a^
37‐48	64	41 (64.1)	23 (35.9)	3.02 (1.21‐7.5)	.02
25‐36	88	45 (51.1)	43 (48.9)	5.14 (2.17‐12.17)	<.001
12‐24	89	57 (64)	32 (48.9)	3.02 (1.26‐7.2)	.01
Gender, n (%)					.73
Girl	132	86 (65.2)	46 (34.8)	Ref	
Boy	160	100 (62.5)	60 (37.5)	1.12 (0.69‐1.81)	
Comorbidity, n (%)					.63
No	120	74 (61.7)	46 (38.3)	Ref	
Yes	172	112 (65.1)	60 (34.9)	0.86 (0.53-1.4)	
Severity, n (%)					
Asymptomatic	16	10 (62.5)	6 (37.5)	Ref	—
Mild-moderate	235	153 (65.1)	82 (34.9)	0.89 (0.31-2.55)	.83
Severe-critical	41	23 (56.1)	18 (43.9)	1.3 (0.4-4.27)	.66
Length of stay (days), median (IQR)	292	3 (1-28)	3 (1-19)	1.02 (0.95-1.08)	.31

a Not applicable.

As shown in Table 3, short stature was the most common among children aged 12‐24 months (n=43, 48.3%), boys (n=77, 48.1%), those with comorbidities (n=82, 47.2%), and those with mild to moderate illness (n=110, 46.8%).

**Table 3.** The correlation between physiological and situational factors with children’s length/height-for-age post–COVID-19.

Variables	Respondents (N=292), n	Growth status			
		Normal stature (n=157)	Short stature (n=135)	OR (95% CI)	*P* value
Age (months), n (%)					
49‐60	51	31 (60.8)	20 (39.2)	Ref	—^a^
37‐48	64	34 (53.1)	30 (46.9)	1.37 (0.65‐2.89)	.41
25‐36	88	46 (52.3)	42 (47.7)	1.42 (0.7‐2.85)	.33
12‐24	89	46 (51.7)	43 (48.3)	1.45 (0.7‐2.92)	.30
Gender, n (%)					.55
Girl	132	74 (56.1)	58 (43.9)	Ref	
Boy	160	83 (51.9)	77 (48.1)	1.18 (0.75‐1.88)	
Comorbidity, n (%)					.64
No	120	67 (55.8)	53 (44.2)	Ref	
Yes	172	90 (52.3)	82 (47.2)	1.15 (0.72‐1.84)	
Severity, n (%)					
Asymptomatic	16	10	6	Ref	—
Mild-moderate	235	125	110	1.47 (0.52‐4.17)	.47
Severe-critical	41	22	19	1.44 (0.44‐4.7)	.55
Length of stay (days), median (IQR)	292	3 (1-28)	3 (1-21)	0.99 (0.94-1.06)	.50

a Not applicable.

According to Table 4, atypical development was most frequently observed among children aged 37‐48 months (n=44, 68.8%), boys (n=111, 69.4%), those with comorbidities (n=121, 70.3%), and those with severe to critical illness (n=32, 78%).

**Table 4.** The correlation between physiological and situational factors with children’s developmental status post–COVID-19.

Variables	Respondents (N=292), n	Developmental status			
		Typical (n=100)	Atypical (n=192)	OR (95% CI)	*P* value
Age (months), n %					
49‐60	51	18 (35.3)	33 (64.7)	Ref	—^a^
37‐48	64	20 (31.3)	44 (68.8)	1.2 (0.55‐2.62)	.65
25‐36	88	32 (36.4)	56 (63.6)	0.96 (0.47‐1.96)	.90
12‐24	89	30 (33.7)	59 (66.3)	1.07 (0.52‐2.21)	.85
Gender, n (%)					.19
Girl	132	51 (38.6)	81 (61.4)	Ref	
Boy	160	49 (30.6)	111 (69.4)	1.43 (0.88‐2.32)	
Comorbidity, n (%)					.06
No	120	49 (40.8)	71 (59.2)	Ref	
Yes	172	51 (29.7)	121 (70.3)	1.64 (1‐2.67)	
Severity, n (%)					
Asymptomatic	16	7 (43.8)	9 (56.3)	Ref	—
Mild-moderate	235	84 (35.7)	151 (64.3)	1.4 (0.5‐3.89)	.52
Severe-critical	41	9 (22)	32 (78)	2.77 (0.81‐9.57)	.11
Length of stay (days), median (IQR)	292	3 (1-26)	3 (1-28)	1.09 (1‐1.19)	.26

a Not applicable.

Multivariate models refined these findings, as presented in Tables 5-7 regarding growth and development status. For weight-for-age, age remained the sole independent predictor. After adjustment, children aged 25‐36 months had approximately five times the odds of being underweight compared to those aged 12‐24 months (*P*<.001), and children aged 49‐60 months had about 3 times the odds. No other factors were retained.

**Table 5.** The final model of dominant factors associated with weight-for-age child growth status post–COVID-19.

Variables	Weight-for-age growth			
	β	OR	95% CI	*P* value
Intercept	–1.682	0.186	—^a^^,^^b^	<.001
Age (months)				
49‐60	Ref	Ref	Ref	—
37‐48	1.104	3.02	1.21–7.5	.02
25‐36	1.636	5.14	2.17‐12.2	<.001
12‐24	1.104	3.02	1.26‐7.2	.01

a The 95% CI is not mentioned because the intercept usually has limited practical interpretation, especially in multivariate models where "all predictors = 0" may not be a meaningful or realistic scenario. Therefore, the focus is generally placed on the predictor variables, as they provide more relevant information regarding associations with the outcome.

b Not applicable.

**Table 6.** The final model of dominant factors associated with length/height-for-age child growth status post–COVID-19.

Variables	Length/height-for-age growth			
	β	OR	95% CI	*P* value
Intercept	–.105	0.9	.34‐3.24	.82
Age (months)				
49‐60	—^a^	Ref	Ref	—
37‐48	.045	1.05	0.34‐3.24	.94
25‐36	–.545	0.57	0.19‐1.81	.35
12‐24	–.028	0.97	0.33-2.85	.96
Gender	–.541			.36
Girl		Ref	Ref	
Boy		0.58	0.18‐1.86	
Age and gender		2.49	—	
37‐48 months and boy	.408			.60
25‐36 months and boy	1.458			.05
12‐24 months and boy	.675			.35

a Not applicable.

**Table 7.** The final model of dominant factors associated with child development status post–COVID-19.

Variable	β	OR	95% CI	*P* value
Intercept	–.021	0.978	—^a^	.94
Comorbidity	.542			.03
No		Ref	Ref	
Yes		1.72	1.05‐2.82	
Length of stay (days)	.095	1.1	1.01‐1.2	.03

a The 95% CI is not mentioned because the intercept usually has limited practical interpretation, especially in multivariate models where "all predictors = 0" may not be a meaningful or realistic scenario. Therefore, the focus is generally placed on the predictor variables, as they provide more relevant information regarding associations with the outcome.

For length-for-age, the final model included an age–gender interaction: boys aged 25‐36 months were significantly more likely to be short-statured than older boys (adjusted odds ratio [aOR] 2.49, *P*=.05). The other age or sex subgroups or any comorbidity or severity variables did not have a significant effect on stature after adjustment.

Regarding developmental status, children with comorbid conditions had significantly higher odds of atypical development (aOR 1.72, 95% CI 1.05‐2.82). Additionally, each extra hospital day slightly increased this risk (aOR 1.10, 95% CI 1.01‐1.20) per day. Age, sex, and illness severity were not significant predictors of development in the adjusted model.

The severity variable was excluded from the final multivariate model as it was not a candidate for retention due to *P* value >.25, exceeding the usual cutoff for inclusion in the multivariate model (as described in Table 4).

## Discussion

### Principal Findings

This study found no significant association between age and child development, contrasting with prior findings linking nutritional status to early developmental outcomes. Galasso & Wagstaff [15] reported a positive correlation between nutrition and development in children under 5 years of age [15], while Shrestha et al [16] emphasized the risks of wasting and underweight status [16]. Although early malnutrition is associated with poor cognitive and motor outcomes [17], our null findings may reflect differences in age distribution, sample characteristics, or developmental assessments.

In contrast to Androutsos et al [9] and Xiao et al [18], who found behavioral issues in children aged 6‐7 years during and after lockdowns, we found no link between age or COVID-19 severity and development. This discrepancy could be due to milder illness in our cohort or differing definitions of severity.

While previous literature often shows gender disparities—such as boys being more prone to stunting due to biological and sociocultural factors [5,8]—we found no significant gender effect. This may be attributed to our sample size, local caregiving practices, or statistical adjustments that controlled for confounding variables.

Although comorbidities such as malnutrition and coronary heart disease increase COVID-19 mortality risk in children [19], our study found no association between these comorbidities and growth or development. This could be due to the low prevalence of those comorbidities (ie, malnutrition and coronary heart disease), milder disease, or differences in comorbidity definitions.

Severe malnutrition is widely linked to developmental delays, with some studies reporting delays in more than >60% of affected children. However, our severity variable was not significant, possibly due to inconsistent classification, low disease severity among children, or the influence of statistical controls diminishing its apparent effect.

Respiratory and neurological impacts of COVID-19, as reported by Bögli et al [20], did not emerge as significant in our cohort—possibly due to the predominance of mild presentation.

Coronary heart disease is known to impair motor development due to chronic hypoxia [21], and febrile seizures in children with epilepsy can lead to neurological damage [22]. These conditions, while important, were infrequent in our population.

Finally, while Ludvigsson [1] argued it was too early to determine if children under 3 years of age are more vulnerable to COVID-19, newborns remain at higher risk due to immature immune systems and lack of maternal antibodies [23].

### Limitations

Discharged patient records were no longer accessible via the electronic health records, and medical records officers were only available until 9 PM daily, leading to delayed data retrieval.

### Conclusions

Age was significantly correlated with child growth. Significant correlations were also found between comorbidities and length of stay and child development. To improve child health outcomes post–COVID-19, comprehensive discharge planning should be provided to families to ensure continuous stimulation, and community health care services should be optimized to offer follow-up care and monitoring for children posthospitalization.

## Abbreviations

aOR: adjusted odds ratio
PDQ: Prescreening Developmental Questionnaire

## References

[1] Ludvigsson JF (2020). Systematic review of COVID-19 in children shows milder cases and a better prognosis than adults. Acta Paediatr.

[2] Dong Y, Mo X, Hu Y (2020). Epidemiology of COVID-19 among children in China. Pediatrics.

[3] Khamsuk A, Whanchit W (2021). Storytelling: an alternative home delivery of English vocabulary for preschoolers during COVID-19’s lockdown in southern Thailand. South African Journal of Childhood Education.

[4] Mulyani I, Wanda D, Agustini N (2021). Dampak situasi pandemi COVID-19 terhadap tumbuh kembang anak [Article in Bahasa Indonesia]. JOTING.

[5] Hockenberry MJ, Rodgers CC, Wilson D (2019). Wong’s Nursing Care of Infants and Children.

[6] Burkhart K, Minnes S, Yamoah O (2022). The effects of COVID-19-related stress among parents and children in Ohio child care programs: a mixed-methods study. Child Health Care.

[7] Ellis WE, Dumas TM, Forbes LM (2020). Physically isolated but socially connected: psychological adjustment and stress among adolescents during the initial COVID-19 crisis. Canadian Journal of Behavioural Science / Revue canadienne des sciences du comportement.

[8] Caputi M, Forresi B, Giani L, Michelini G, Scaini S (2021). Italian children’s well-being after lockdown: predictors of psychopathological symptoms in times of COVID-19. Int J Environ Res Public Health.

[9] Androutsos O, Perperidi M, Georgiou C, Chouliaras G (2021). Lifestyle changes and determinants of children’s and adolescents’ body weight increase during the first COVID-19 lockdown in Greece: The COV-EAT Study. Nutrients.

[10] Bloise S, Isoldi S, Marcellino A (2022). Clinical picture and long-term symptoms of SARS-CoV-2 infection in an Italian pediatric population. Ital J Pediatr.

[11] Benmerzoug M, Djoudi B, Debbache A (2022). Impact of COVID-19 lockdown on children’s health in North Africa. Matern Child Health J.

[12] Araújo L de, Veloso CF, Souza M de C, Azevedo J de, Tarro G (2021). The potential impact of the COVID-19 pandemic on child growth and development: a systematic review. J Pediatr (Rio J).

[13] Panda PK, Gupta J, Chowdhury SR (2021). Psychological and behavioral impact of lockdown and quarantine measures for COVID-19 pandemic on children, adolescents and caregivers: a systematic review and meta-analysis. J Trop Pediatr.

[14] Meherali S, Punjani N, Louie-Poon S (2021). Mental health of children and adolescents amidst COVID-19 and past pandemics: a rapid systematic review. Int J Environ Res Public Health.

[15] Galasso E, Wagstaff A (2019). The aggregate income losses from childhood stunting and the returns to a nutrition intervention aimed at reducing stunting. Econ Hum Biol.

[16] Shrestha ML, Perry KE, Thapa B, Adhikari RP, Weissman A (2022). Malnutrition matters: association of stunting and underweight with early childhood development indicators in Nepal. Matern Child Nutr.

[17] Kang Y, Aguayo VM, Campbell RK, West KP (2018). Association between stunting and early childhood development among children aged 36-59 months in South Asia. Matern Child Nutr.

[18] Xiao H, Liu Q, Mei H (2022). Behavioral problems of pediatric patients recovered from COVID-19 in Wuhan, China. Acta Psychol (Amst).

[19] Pudjiadi AH, Putri ND, Sjakti HA (2021). Pediatric COVID-19: report from Indonesian Pediatric Society Data Registry. Front Pediatr.

[20] Bögli J, Güsewell S, Strässle R, Kahlert CR, Albrich WC (2023). Pediatric hospital admissions, case severity, and length of hospital stay during the first 18 months of the COVID-19 pandemic in a tertiary children’s hospital in Switzerland. Infection.

[21] Pambudi J, Dhamayanti M, Kuswiyanto RB (2019). Perbedaan status perkembangan dan pertumbuhan anak dengan penyakit jantung bawaan sianotik dan non-sianotik [Article in Indonesian]. Sari Pediatri.

[22] Nikbakht F, Mohammadkhanizadeh A, Mohammadi E (2020). How does the COVID-19 cause seizure and epilepsy in patients? The potential mechanisms. Mult Scler Relat Disord.

[23] Dhochak N, Singhal T, Kabra SK, Lodha R (2020). Pathophysiology of COVID-19: why children fare better than adults?. Indian J Pediatr.

